# Copy number variants and selective sweeps in natural populations of the house mouse (*Mus musculus domesticus*)

**DOI:** 10.3389/fgene.2014.00153

**Published:** 2014-06-03

**Authors:** Jarosław Bryk, Diethard Tautz

**Affiliations:** Max Planck Institute for Evolutionary BiologyPlön, Germany

**Keywords:** CNV, selective sweeps, mice, CGH microarray, natural populations

## Abstract

Copy–number variants (CNVs) may play an important role in early adaptations, potentially facilitating rapid divergence of populations. We describe an approach to study this question by investigating CNVs present in natural populations of mice in the early stages of divergence and their involvement in selective sweeps. We have analyzed individuals from two recently diverged natural populations of the house mouse (*Mus musculus domesticus*) from Germany and France using custom, high–density, comparative genome hybridization arrays (CGH) that covered almost 164 Mb and 2444 genes. One thousand eight hundred and sixty one of those genes we previously identified as differentially expressed between these populations, while the expression of the remaining genes was invariant. In total, we identified 1868 CNVs across all 10 samples, 200 bp to 600 kb in size and affecting 424 genic regions. Roughly two thirds of all CNVs found were deletions. We found no enrichment of CNVs among the differentially expressed genes between the populations compared to the invariant ones, nor any meaningful correlation between CNVs and gene expression changes. Among the CNV genes, we found cellular component gene ontology categories of the synapse overrepresented among all the 2444 genes tested. To investigate potential adaptive significance of the CNV regions, we selected six that showed large differences in frequency of CNVs between the two populations and analyzed variation in at least two microsatellites surrounding the loci in a sample of 46 unrelated animals from the same populations collected in field trappings. We identified two loci with large differences in microsatellite heterozygosity (*Sfi1* and *Glo1*/*Dnahc8* regions) and one locus with low variation across the populations (*Cmah*), thus suggesting that these genomic regions might have recently undergone selective sweeps. Interestingly, the *Glo1* CNV has previously been implicated in anxiety–like behavior in mice, suggesting a differential evolution of a behavioral trait.

## Background

Copy–number variants (CNVs) have become an appreciated type of variation in the genomes of humans and mice. While there is no common definition of what a CNV is, it is usually applied to segments of DNA in size from about 1 kb to several megabases that vary in number in relation to a reference genome (Feuk et al., 2006). Since technological advances in microarray technologies and sequencing allowed genome–wide investigation of this type of structural variation in humans (Redon et al., 2006) and mice (Cutler et al., 2007; Graubert et al., 2007), it has become apparent that CNVs are (a) numerous and ubiquitous (b) variable in size, including very large ones and (c) highly mutable. For example, across 41 strains of inbred mouse lines there were on average 51 CNVs (range 15–106 CNV per genome) with an average size of 183 kb (amplification) and 204 kb (deletion) (Cutler et al., 2007) per genome. Analysis of genealogies of the substrains of the B6 mouse strain indicated that CNVs arise spontaneously in every 46–139 newborn inbred mice, indicating in some cases a mutation rate comparable to that of microsatellites (Egan et al., 2007).

Despite the fact that CNVs affect such a large fraction of the human and mouse genome, relatively few phenotypic effects are known for the CNVs. It is likely that most of the CNVs are either neutral or slightly deleterious and have not yet been removed from the genome by purifying selection (Hastings et al., 2009). Still, there are cases where CNVs contribute to fitness of an organism. For example, in human populations with a history of eating starch, a gene encoding starch–digesting enzyme amylase has more copies and produces more salivary amylase than in populations with no starch–eating history, with population genetic evidence for spread of the multiplied DNA segment in starch-eating populations (Perry et al., 2007). Similarly, the pancreatic *Amy2b* gene cluster in dogs was subject to copy number increase as a response to more starch-rich diets in dog breeds (Axelsson et al., 2013). Further, humans with more copies of a ligand that binds the same receptor as HIV-1 are less susceptible to infection (Gonzalez et al., 2005). More generally, CNVs were also shown to affect gene expression: in human lymphoblastoid cell lines, CNVs accounted for about 18% of variation in gene expression (Stranger et al., 2007). In inbred and wild mouse populations CNVs were found to account for 38–73% of variation in gene expression in various tissues (Henrichsen et al., 2009). While in both of these studies increase of expression level highly but not completely correlates with an increase in copy number, human tumors are characterized by indirect and complex effects of CNVs on gene expression (Lee et al., 2008).

Given the observations that CNVs are numerous, mutable, large enough to encompass whole gene regions and already known to affect gene expression and overt phenotypes, we reasoned that CNVs may play an important role in early adaptations, facilitating rapid divergence of populations. We therefore decided to more systematically investigate CNVs present in natural populations of mice in the early stages of divergence and their potential role in adaptations.

## Results

### Experimental design

**Figure 1:**
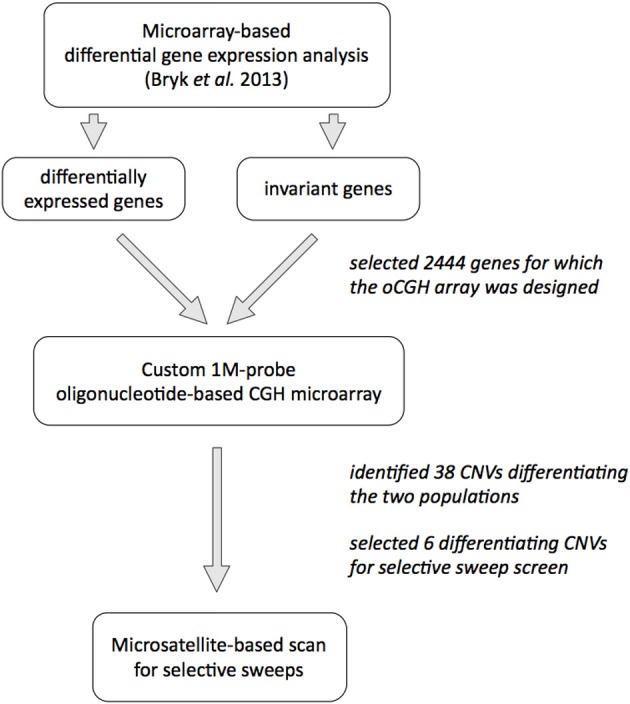
**Overview of the experimental design**.

Our subject are allopatric populations of the house mouse *Mus musculus domesticus*, from the Massif Central in France and around Cologne/Bonn area in Germany that diverged less than 3000 years ago (Cucchi et al., 2005); details of the populations and trappings are published in Ihle et al. (2006). We previously analyzed gene expression patterns in animals from both populations (Bryk et al., 2013) and decided to use gene expression differences as a proxy for identification of CNVs. Our hypothesis was that since CNVs are known to affect gene expression levels, we may enrich for CNVs by using genes for which we detected differences in gene expression between the two populations. To test the alternative hypothesis that gene expression differences are not a good predictor for CNVs, we also used genes with invariant expression across the two populations in the screen. A schematic overview of the experimental approach is shown on Figure 1.

To investigate CNVs on a genome–wide scale, we designed a custom, high–resolution oligonucleotide–based comparative genome hybridization (CGH) microarray that contained 950 k 60 mer probes covering about 164 Mb of the mouse genome (on average, one probe every 170 bp). The probes were placed in the genomic regions of 2444 genes and 10 kb up—and downstream sequences from each gene. One thousand eight hundred and sixty one of these genes were differentially expressed between the populations in at least one of three tissues tested (brain, liver, testis), while 583 genes were invariant in between the populations (see Bryk et al., 2013 and Methods for the details; list of all genes on the CGH array is provided in Table S1).

We used tail DNA from five unrelated males from each population and hybridized it to the microarray along with tail DNA obtained from a male from the reference strain C57Bl/6. The wild males used in this experiment were brothers of animals used for the gene expression analysis (Bryk et al., 2013). We defined a copy–number difference as at least three consecutive probes with an absolute log_2_ ratio > 0.5 relative to the reference genome.

### There are numerous small CNVs in all samples tested

**Figure 2:**
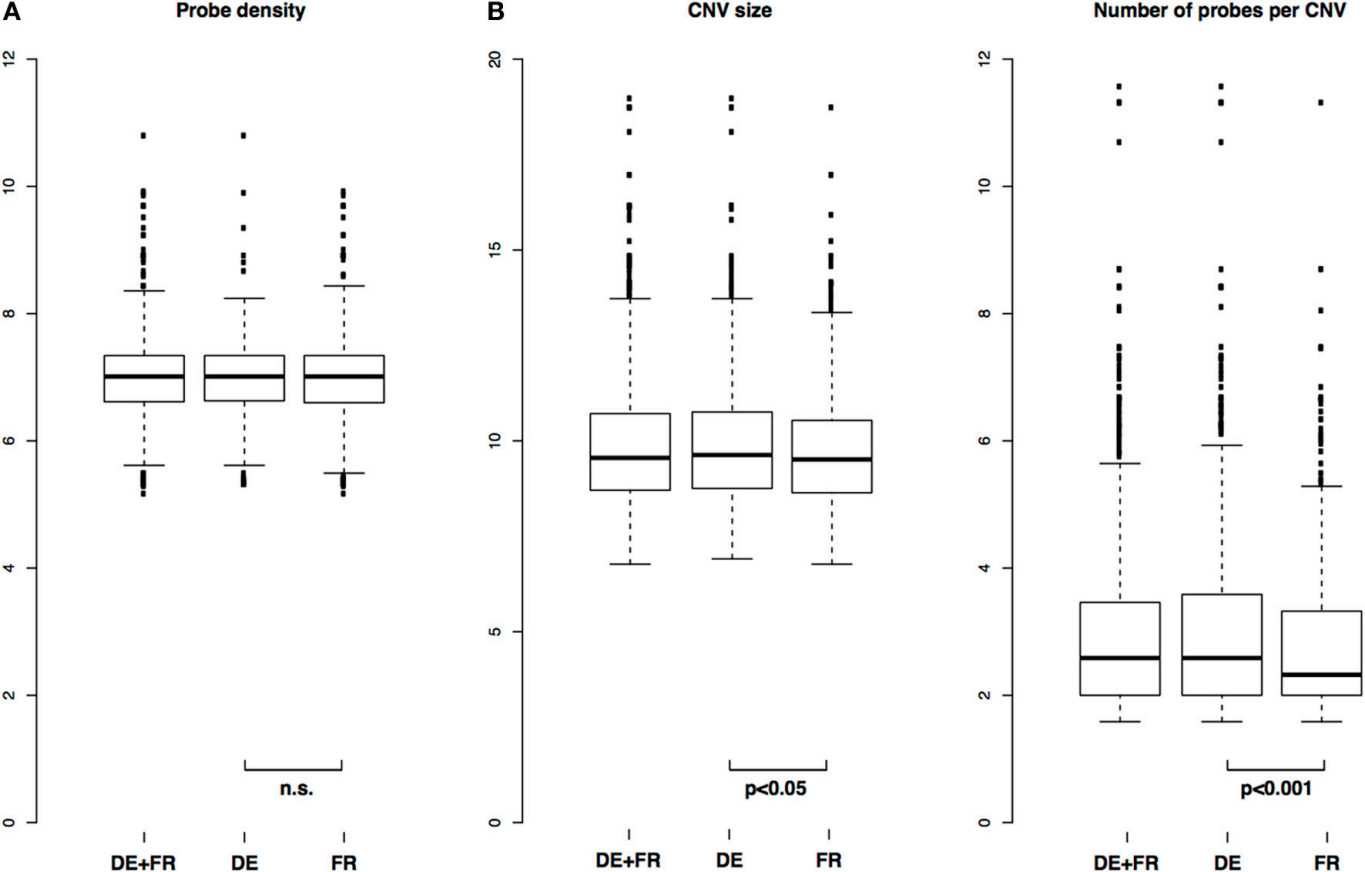
**Distribution of CNV length, number of probes and probe density in CNVs**. **(A)** Comparison of distributions of probe densities in all samples and in each population separately and **(B)** Comparison of distributions of CNV size and number of probes per CNV in all samples and in each population separately. *P*-values from two-sample Kolmogorov-Smirnov test.

We identified 1868 CNVs across all 10 samples tested (1015 CNVs if each CNV is counted only once) (Table S2). There were between 85 and 336 CNVs per genome, well within ranges reported before in mice (i.e., Cutler et al., 2007). The distributions of probe density in CNVs (defined as CNV length divided by number of probes in that CNV) were not different between the two populations, indicating a similar power to detect CNVs in the two groups (two-sample Kolmogorov-Smirnov test, *p* = 0.16) (Figure 2A).

Across all samples, median size of a CNV was 751 bp with mean 3526 bp, and median number of probes per CNV was 6, with mean 21. In the German population, median and mean CNV size was 791 bp and 4655 bp, respectively, and in the French population 730 bp and 2388 bp, respectively. In terms of median and mean number of probes per CNV, the German population was 6 and 28, respectively, and the French 5 and 14, respectively. The distributions of CNV size and number of probes per CNV between the two populations were significantly different between the two populations, with *p* = 0.04 (CNV size) and *p* = 0.0004 (number of probes per CNV) (two-sample Kolmogorov-Smirnov test). Overall and population-specific distributions are depicted in Figure 2B.

We found no differences in number of CNVs detected in the two populations, with total number of CNVs in the German population 938 and in the French 930 (*p* = 0.84, Mann-Whitney *U*-test). Overall, we identified more deletions than amplifications: 1449 vs. 419 (*p* < 0.001, Mann-Whitney *U*-test), but there were no differences between number of amplifications or deletions in each group: 223 vs. 196 amplifications and 715 vs. 734 deletions between the German and the French populations, respectively (*p* = 0.45, Fisher's exact test).

To assess a potential functional impact of the discovered CNVs, we analyzed the chromosomal locations of the CNVs by cross-checking them with the positions of introns and exons (including 3'- and 5'-UTR exons) from the UCSC Genome Browser using the Ensembl genes database (see Methods). The CNVs overlapped 4068 of these elements in 414 genes by at least 10% of the length of the CNV. 3564 of these elements (88%) were introns, 316 (7.8%), 130 (3.2%), and 58 (1.4%) were exons, 3'-exons and 5'-exons, respectively. The median fraction of length of exons overlapped by CNVs was 80%, of 3'-exons 57% and 5'-exons 94%, with introns only 5% (Table S3).

To elucidate whether the 424 genic regions with CNVs detected in the two populations share a common function or cellular location, we performed a gene ontology (GO) analysis for overrepresentation of CNV-affected genes' GO category among all the genes that we tested on the CGH array. We found six cellular component categories that were significantly overrepresented (hypergeometric test, *p* < 0.05 after Benjamini-Hochberg FDR correction): three of them involved synaptic structures and the others are involved in cell and nuclear membranes. The details of the GO analysis are presented in Table S4.

### Gene expression differences do not associate with copy–number variation

Our data allowed us to explore the relationship between CNVs and gene expression changes in some detail, bearing in mind the limitations of our study: gene expression and CNV data came from different microarray platforms, genomes and tissues, and we pre-selected the genes we screened for CNVs. Since our gene expression data was acquired on two platforms (Bryk et al., 2013), we performed a series of Spearman's rank correlation tests on data from both platforms and in each tissue separately, looking for cases with consistent correlations between platforms. In all tissues and platforms, we observed highly significant but small positive correlation between differences in gene expression as measured by *p*-values and CNV size (all *p* < 2 × 10^−6^ and all rho < 0.22). We were unable, however, to find consistent correlations between differentiating CNV sizes (see below) and *p*-values or t statistics values, or when we restricted the comparison to genes differentially expressed between populations (data not shown).

Finally, when we compared the number of CNV–containing genes that were differentially expressed between and invariant across the two populations, we found no significant differences (Fisher's exact test, *p* = 0.50): there were 60 CNVs detected among the 506 invariant genes (12.7%) and 248 CNVs among 1861 differentially expressed genes (15.0%). This finding also held when we restricted the data only to CNVs larger than 10 kb (data not shown).

### Few CNVs differentiate the two populations

We reasoned that if there were any CNVs contributing to the divergence of the two populations, the number of copies of such loci would be different between the two groups. Since CGH data is not reliable enough to quantify copy number of DNA fragments (Pozhitkov et al., 2014), to select putatively differentiating CNVs for subsequent analyses, we chose those CNVs that were amplified or deleted in at least four samples in one population and in no more than one sample in the other population, with significant difference in number of called amplifications or deletions in each group (hypergeometric test, *p* < 0.05). We found 38 such CNVs, which are summarized in Table S5. They range from a 274 bp deletion found in all German samples but in none of the French in an intron of the amylase 1 gene (*Amy1*) to a 0.5 Mb-long amplification that includes several genes (*Btbd9, Glo1* and *Dnahc8*) in all German samples and in only a single French sample.

**Figure 3:**
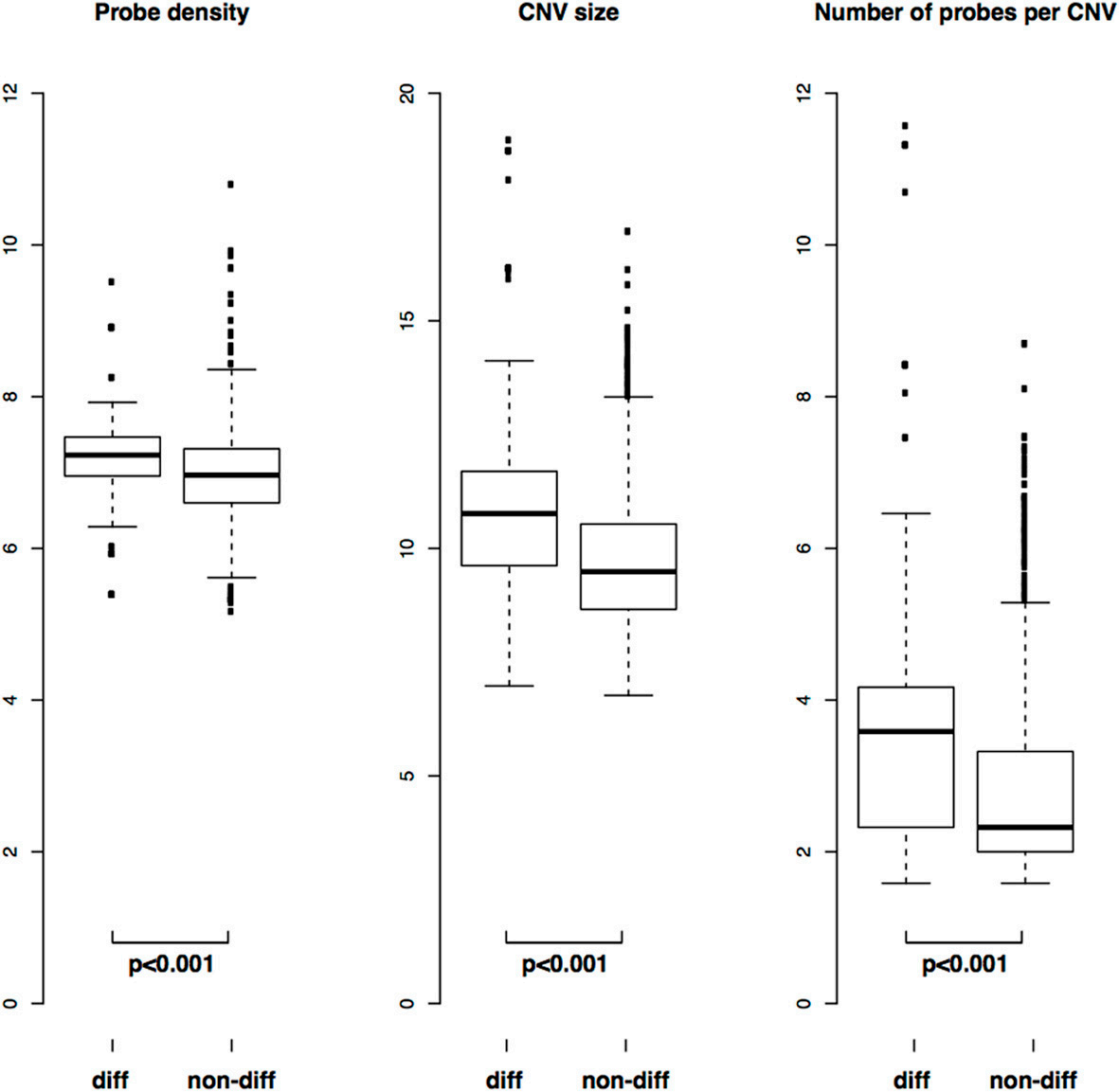
**Comparison of distributions of CNV length, number of probes and probe density in differentiating (“diff”) vs. non-differentiating (“non-diff”) CNVs**. *P*-values from two-sample Kolmogorov-Smirnov test.

The average size of the differentiating CNVs is about 12 kb, with median size about 1.5 kb, with 72 and 9 probes affected, respectively. The distributions of CNV sizes, number of probes and probe density are significantly different for differentiating vs. non-differentiating CNVs (two-sample Kolmogorov-Smirnov test, *p* < 0.001), with differentiating CNVs being larger, containing more probes and having higher probe density (Figure 3).

Five of the differentiating CNVs affect either the whole gene region or a part of a gene including introns and exons, while the rest is located in introns or between genes. Twenty six of the differentiating CNVs (68%) were associated with differences in gene expression. However, there was no association between differentiating CNVs and direction of gene expression difference: among the 38 CNVs, 12 corresponded to no change in gene expression, 11 showed change in opposite direction to expected (i.e., amplification resulted in lower gene expression), 14 in the expected direction, and 1 corresponded to opposing changes in gene expression in different tissues (binomial test for number of CNVs with expected and opposite direction of gene expression change, *p* = 0.69) (Table S5). In addition, we found no GO ontology categories overrepresented among only the differentiating CNVs.

### CNVs show differences in microsatellite heterozygosity around the differentiating loci

To investigate whether the CNVs differentiating the two populations could be under positive selection in one of the populations, we performed a scan for microsatellite length heterozygosity around the respective loci using lnRH statistic and software developed by (Schlötterer, 2002; Dieringer and Schlötterer, 2003). Of the 38 differentiating CNVs, we picked all three that affected exons or whole gene regions (*Btbd9/Glo1/Dnahc8, Gm7210, Sfi1/Pisd-ps1*), as well as the two largest amplified CNVs located in an intron or in an intergenic region (*Cmah* and *Dgke*). In addition, we also picked the small intronic CNV in *Amy1* gene.

We performed the scan using a panel of 46 unrelated individuals from each population that originally came from field trappings (Ihle et al., 2006). We PCR–amplified between two and four microsatellite loci in a 10 kb region up–or downstream of each aberrant loci (15 kb in the case of *Amy1*) using CGH data to precisely delineate aberrant positions. We compared heterozygosity of their repeat lengths in the two populations relative to a panel of 64 neutral microsatellites identified previously by Teschke et al. (2008). The location and primer sequences for microsatellite amplifications are given in Table S6.

We found that microsatellites around CNVs affecting *Sfi1/Pisd-ps1* and *Btbd9/Glo1/Dnahc8* regions show significant differences (lnRH statistics, |*z*| > 2, which corresponds to *p* < 0.01) in heterozygosity between the two populations, and the CNV in the *Cmah* gene region show consistent and very low microsatellite heterozygosity values in both populations. The *Amy1* CNV showed inconsistent changes between populations and stages, while *Gm7120* and *Dgke* showed no differences (Table S7).

## Discussion

Here we document a pattern of copy-number variants in 2444 genes in high resolution in two recently diverged allopatric populations of wild *Mus musculus domesticus.* Technically, our study follows the work of (Cutler et al., 2007), which used Agilent's off-the-shelf oligonucleotide-based comparative genome hybridization microarrays to probe multiple mouse genomes for CNVs. While using the same technology, we designed a microarray with much higher probe density, allowing us to probe genomes for much smaller CNVs than those previously published by Cutler et al. (2007), albeit only in a fraction of the genome.

It is worth noting that the genomes of *Mus musculus domesticus* investigated in this work are different from the reference genome of the C57Bl/6 mouse (Yang et al., 2007; Didion and Villena, 2013). We argue, however, that potential sequence variation between the wild and reference genomes should not affect our results for the following reasons: (a) C57Bl/6 is a mosaic genome, but pre-dominantly (92%) derived from *Mus musculus domesticus* (Yang et al., 2007) and (b) when analyzing gene expression data we checked for differences in hybridization performance due to sequence variation (gene expression probes were also designed based on C57Bl/6 genome), and found negligible effects of potential sequence differences on probe hybridization (Bryk et al., 2013). However, we cannot exclude that C57Bl/6 genome fragments derived from more distantly related species had an impact on hybridization with wild genomes (Didion and Villena, 2013).

A second consideration regarding CNVs numbers and size identified in this study is a definition of CNV and quantification of copy numbers in each sample. We defined CNV as a group of at least three consecutive probes, each with absolute log_2_ ratio larger than 0.5. While arbitrary, this is a choice shared by other custom oligonucleotide-based CGH designs in non-model species (i.e., Wang et al., 2014), and we argue that the choice of relatively small number of three consecutive probes was justified given our very high probe density design. In addition, Figure 2 and Table S2 demonstrate that vast majority of the CNVs contained more than 3 probes, indicating little effect of that definition on global CNV discovery.

Traditionally, CGH arrays are not used to asses copy number directly, in part due to its relatively low resolution, and the copy number changes need to be quantified using quantitative PCR or fiber-FISH (Perry et al., 2007). Also, there is a substantial noise in standard arrays that would require proper assessment and calibration to directly infer copy numbers (Pozhitkov et al., 2014). CNVs assessed by CGH arrays are therefore usually identified as amplifications and deletions only (Wineinger et al., 2008), as hybridization performance is not reliable enough to quantify copies of DNA segments directly (although large log_2_ ratios may give some indication about the degree of change). We therefore chose number of called CNVs per population as indication of population differentiation, acknowledging that we are likely to underestimate the number of differentiating CNVs between the populations, as we ignore situations where e.g., all French samples had twice as many copies of a DNA fragments as C57Bl/6, and all German samples had four times as many copies as C57Bl/6.

Nevertheless, we still were able to identify many small CNVs that could have not been detected before due to lack of resolution of off-the-shelf CGH platforms; indeed, our distribution of CNV sizes falls almost exactly in the resolution gap visualized by Cutler et al. (2007) in their Figure 3. More recent approaches to CNV discovery using high-throughput sequencing also find extensive sub-2 kb copy-number variation in the mouse genome (Yalcin et al., 2011). This finding further emphasizes the ubiquitous nature of CNVs across all sizes, from several hundred bases up to a megabase range.

Similar to previous studies employing CGH, we found an excess of deletions vs. amplifications. This is a common finding in CGH studies (i.e., Cutler et al., 2007; Graubert et al., 2007), likely because a deletion is easier to detect as it is an absence of a signal, whereas amplification often results in only a fractional increase of a signal. We also note that the higher the resolution of CGH studies, the higher the proportion of deletions are found (Carter, 2007).

Unlike previous studies that analyzed impact of CNVs on gene expression, we did not find any convincing relationship between gene expression differentiation between the populations and CNVs. The only consistent and significant correlation, between *p*-values and CNV size, was positive but very weak (biggest rho < 0.22, Spearman's rank correlation test). This suggests, counter-intuitively, that *p*-values increase (gene expression differences diminish) with increasing CNV length. The nature of this relationship is unclear given lack of any consistent correlation in t statistics values or probe density, or when differentially expressed genes or when only large CNVs are analyzed (data not shown). This scarcity of gene expression and CNVs relationship is underscored by perhaps our most surprising finding: no difference in fraction of CNV-containing genes identified among the differentially expressed or invariant genes. These findings can be explained by the overall small size of CNVs identified in this study compared to previous work (Cutler et al., 2007; Henrichsen et al., 2009; Cooper et al., 2011). Since an average CNV detected in this work is about two orders of magnitude smaller than in previous studies, so is likely to be its impact on phenotypes, including gene expression, as previous studies suggested a positive correlation between CNV size and its phenotypic impact (Cooper et al., 2011). Sequence–based studies that identified sub-1 kb structural variants in mouse genome also find very limited influence of them on gene expression and other phenotypes (Yalcin et al., 2011). Due to the special design of our CGH array, we were unable to test indirect relationships between CNV and neighboring genes or genes located elsewhere in the genome (Lee et al., 2008; Henrichsen et al., 2009).

**Figure 4:**
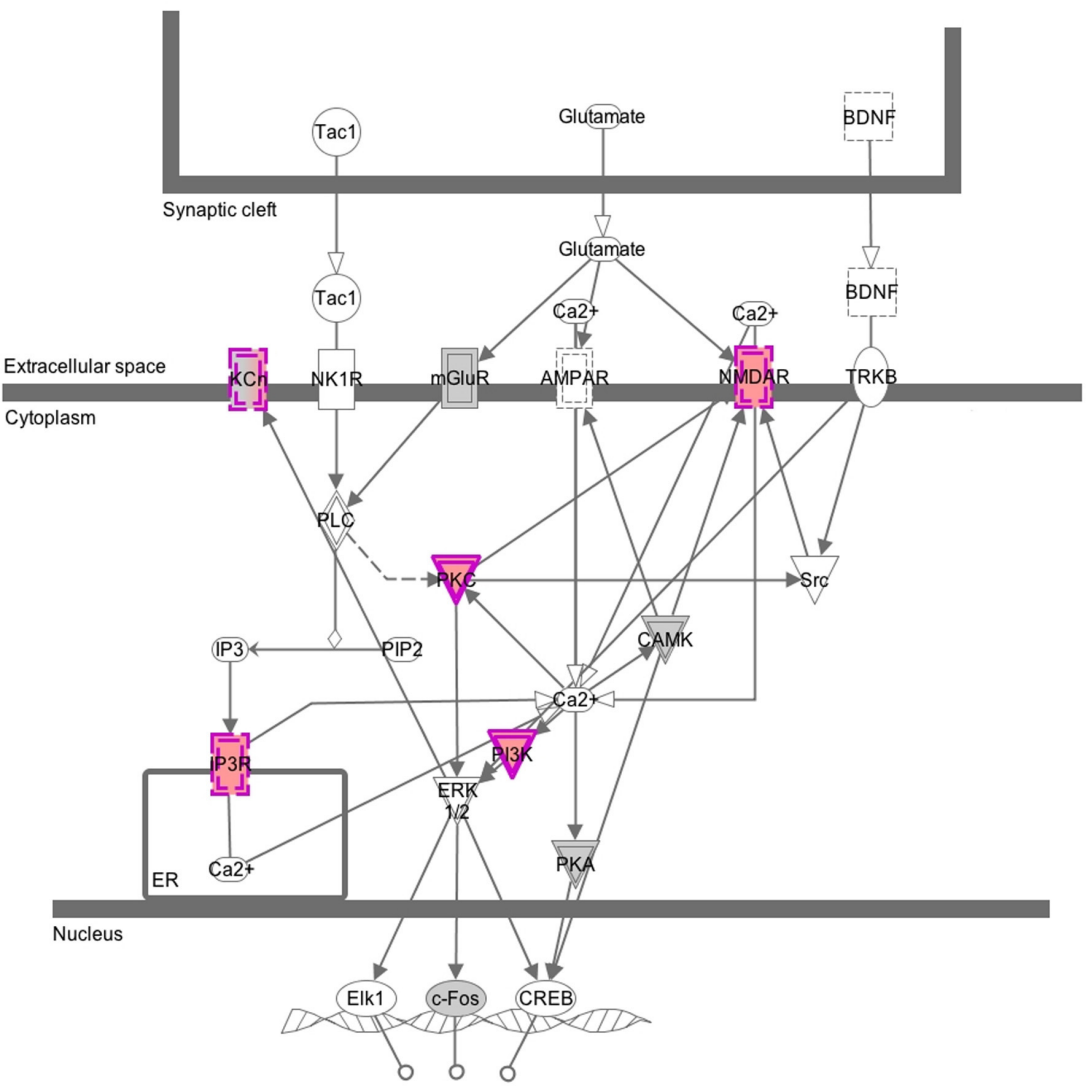
**Graphical overview of the Canonical Pathway analysis**. This image was generated by Canonical Pathways analysis and shows genes that belong to the category of neuropathic pain signaling in dorsal horn neurons. Nodes in gray and purple depict genes from the CGH array submitted for the analysis: in gray, genes without CNVs and in purple, genes with CNVs. Solid lines around a node depict a kinase and dashed lines depict an ion transporter. Double lines indicate complex of several subunits.

While we expected to identify many CNVs in our samples, we found it surprising that they affected genes involved in synaptic structures and cellular membranes of Gene Ontology categories. This finding prompted us to check the genes with CNV in a pathway enrichment analysis using Ingenuity's Canonical Pathways software. We found out that the genes with CNVs are overrepresented in synaptic neuropathic pain signaling in dorsal horn neurons, where several genes (*KCh, NMDAR, PKG, IP3R*, and *PI3K*) contribute to activation of the transcription factors *Elk1, c-Fos*, and *CREB*. This in turn is consistent with our findings from gene expression analysis (Bryk et al., 2013), where differentially expressed genes were overrepresented in the TRANSFAC category of *CREB* family of transcription factors. However, our choice of loci for analysis is evidently biased toward genes that we had identified in the previous expression study. Still, the CNV analysis provides a line of evidence suggesting that the synaptic signaling in the populations tested harbors changes on DNA and transcriptome levels (overview of the Canonical Pathway analysis is shown in Figure 4). However, it is worth emphasizing here that these are CNVs found in both populations; when we ran the GO or Canonical Pathway analysis only on CNVs that differentiate the two groups, we found no significant enrichment of genes from any GO category or pathway tested. It is currently unclear whether the synaptic and membrane structures-related genes that seem prone to harbor CNVs in our populations have any functional significance.

To detect potential functional significance of the detected CNVs, we concentrated our efforts on regions that differ in CNV frequency between the populations. Of the 38 regions significantly different in copy number, the vast majority affects intron and intergenic regions, and only three regions contain exons or whole genes. We reasoned that the latter changes are most likely to have a functional effect. To these three regions we added the two largest CNVs located in an intron and in an intergenic region (*Cmah* and *Dgke*, each ~3 kb amplification in the 4 samples from the German population), as well as *Amy1* (~300 bp deletion in all German samples). We picked *Amy1* because (a) it was one of the highest differentially expressed genes in the brain between the two populations (Bryk et al., 2013) (b) *Amy1* was previously shown to be copy-number variable and involved in adaptation to high-starch content in food in human populations (Perry et al., 2007) (c) previous analyses in our group indicated that the whole amylase gene cluster may be a locus of large introgression between *M. m. musculus* and *M. m. domesticus* populations (Staubach et al., 2012).

Microsatellites are commonly used in scans for selective sweeps due to their high polymorphism and mutagenicity (Schlötterer, 2003; Ellegren, 2004), both characteristics particularly important in our case, where the analyzed populations diverged only at most 3000 years ago (Cucchi et al., 2005). Past analyses of microsatellite diversity in populations of *M. m. domesticus*, including those used in this study, suggested restricted gene flow between the French and German populations and stable demographic structure (Ihle et al., 2006). Crucially, whole-genome microsatellite scans for selective sweeps done by Teschke et al. (2008) in the same populations identified a set of reference microsatellites for which the two populations do not differ in heterozygosity. We compared variation in that set to heterozygosity values obtained from microsatellites located up- and/or downstream of the candidate CNV regions. For the two out of six regions tested we found significant differences in heterozygosity between the two populations: reduced heterozygosity in the French samples for the *Sfi1* region (deleted in this population) and reduced heterozygosity in the German samples for the *Dnahc8* region (amplified in this population). Moreover, heterozygosity values for the *Cmah* CNV were very low across the two populations. This may either indicate a potential selective sweep in both populations, or parallel sweeps for two different nucleotide variants (a situation that the method is not able to identify). The situation for the *Amy1* CNV locus is unclear, with one of three microsatellites tested showing highly significantly reduction in heterozygosity in the French samples (where there is a deletion in all individuals tested), and two other microsatellites showing no significant changes in the two populations.

This is to our knowledge the first study that tried to systematically investigate potential selective sweeps on copy-number variable loci in natural mice populations. While the number of samples and loci tested is low, we demonstrate the feasibility of establishing a relationship between CNVs and signatures of selective sweep and open this line of investigation to hundreds of CNVs identified here with more high-throughput methods. Ultimately, however, such scans are just the beginning: further investigation will determine whether the three loci identified here as CNV–containing regions under selective sweep have any effect on the animals' phenotypes and potentially on their fitness as well. From this perspective, we find it encouraging that the copy-number variation in one of our candidate loci that contains *Glo1* gene was shown to affect mouse behavior. Animals with higher copy number of *Glo1* exhibited increased anxiety (Williams et al., 2009) and it is tempting to speculate that this might be behavioral change of adaptive significance in the wild.

## Methods

### Animal capture and tissue collection

We bred F1 offspring of wild-caught mice from each of the two populations in the laboratory. Parental mice were caught in the Massif Central in France and in the Cologne/Bonn area in Germany (see Results and Ihle et al., 2006). For the CGH experiment, we used tail DNA isolated from five unrelated 12-week-old males from each population, the same animals used in Bryk et al. (2013). For the microsatellite screen, we used DNA from tail clippings from 46 unrelated animals from each population from the same trapping areas. All animals were sacrificed with CO_2_ and dissected at the same time of day. All animal work was registered under number V312-72241.123–34 (97-8/07) and approved by the ethics commission of the Ministerium für Landwirtschaft, Umwelt und ländliche Räume on 27.12.2007.

### Array and sample preparation and data aquisition

DNA was isolated from 0.5 cm tail clippings using Qiagen DNeasy Blood and Tissue kit (Qiagen, Hilden, Germany) and its quality assessed using NanoDrop (Thermo Fisher Scientific, Waltham, USA) and agarose gel electrophoresis. DNA was labeled and hybridized to custom 1 M probe CGH microarray from Agilent (Agilent Technologies, Santa Clara, USA; microarray design ID 28699) according to manufacturer's instructions in G4410-90010 CGH Enzymatic Protocol version 6.3, using flipped design with Cy5 dye used to label the reference genome.

The genes on the custom CGH array were selected from genes analyzed in Bryk et al. (2013), where we performed gene expression microarray analysis in brain, liver and testis of the mice on two microarray platforms, from Affymetrix and Agilent. For the differentially expressed genes, we selected genes differentially expressed in at least one tissue with *p* < 0.05 and FDR < 0.1 in both platforms. For the invariant genes, we divided genes into quantiles based on expression level and then selected 50 genes with lowest coefficient of variation across all samples in each tissue in each quantile. Seventy-seven genes present in both lists (i.e., genes differentially expressed in one tissue but invariant in another) were removed from the analysis of CNV enrichment among differentially expressed or invariant genes.

The arrays were processed in two batches over two days, with 4 and 6 arrays per batch. Following data acquisition, we observed a difference in signal quality between the two batches. dLRsd (derivative log ration spread) algorithm score, which measures probe- to- probe noise, was increased in 4 samples in the first batch, 2 from each population (see Agilent Genomic Workbench 6.5 User Guide page 529). We attribute this difference in signal quality to locally changing ozone concentrations (Fare et al., 2003), as for technical reasons, part of our hybridization protocol took place outside of the ozone-filtered laboratory. We believe, however, that this effect does not affect our results for the following reasons: each CGH array sample is self-contained (it is a two color design) and lower signal quality (increased noise) makes our findings more conservative, as we may observe fewer consistent consecutive probe changes across samples. In addition, in attempt to protect from potential ozone influence, we flipped the array design, labeling the reference genome with Cy5. If the dye was affected by ozone, it should affect probes similarly across the array. Lastly, this effect affected both populations equally.

Design of the custom microarray required us to combine a set of 11,000 probes distributed across the whole genome with about 950,000 densely-spaced probes covering only the selected gene regions. Because the sparsely spaced probes in result spanned very large regions, we excluded them from called CNVs by imposing a limit of probe density (CNV length divided by number of probes) of at least one probe per 2 kb in the called region. This filter removed 11 putative CNVs, none of which would qualify for our differentiating CNV criteria.

### Data analysis

Data were analyzed using Agilent's Genomic Workbench software with the ADM-2 CNV-detecting algorithm with fuzzy zero turned on and threshold set to 10. CNV was defined as three consecutive probes with absolute log_2_ ratio > 0.5. The resulting data were imported into R (Gentleman et al., 2005) for subsequent data manipulation (size and probe number distributions, gene content and annotation).

Overlap with known Ensembl exons and introns was done using the bedtools intersect function (https://github.com/arq5x/bedtools2) on the list of identified CNVs and the list of exons and intros exported from the UCSC Genome Browser (Karolchik et al., 2004). We required each CNV to overlap a given feature by at least 10% of CNVs length. Please note that due to the fact that the export of genomic features uses different criteria than the Agilent Genomic Workbench annotation engine, the lists of genes that contain CNVs from Agilent and from UCSC are slightly different. We used Agilent's annotation in all cases except for this overlap analysis.

GO analysis was performed using WebGestalt online software (Wang et al., 2013), requiring at least 5 genes in a GO category and *p* < 0.05 after FDR correction. Pathway enrichment analysis was performed with Ingenuity Pathway Analysis software's right-tailed Fischer's exact test for overrepresentation of genes in Ingenuity's Canonical Pathways. The input file was a list of all genes on the CGH microarray with values “0,” “0.5,” and “1” assigned to genes with no CNVs, genes with CNVs, and genes with CNVs that differentiate the populations, respectively.

### Microsatellites and selective sweeps

Genomic DNA regions up- and downstream of CNVs were selected using the UCSC Genome Browser (Karolchik et al., 2004). Microsatellites and PCR primers were identified and selected using msatcommander version 1.05 (Rozen and Skaletsky, 1999; Faircloth, 2008; https://code.google.com/p/msatcommander/). We searched for di- or tri-nucleotide motifs of 5–15 repeats only. PCR primers were labeled with FAM or HEX dyes and PCR reactions were run in multiplex using the Qiagen multiplex PCR kit. PCR products were analyzed using the ABI Gene Mapper software version 4.0 (Life Technologies, Carlsbad, USA). Microsatellite length heterozygosity was analyzed with lnRH statistics and software by (Schlötterer, 2002; Dieringer and Schlötterer, 2003).

## Author contributions

Jarosław Bryk and Diethard Tautz designed the experiments, analyzed the data and wrote the manuscript; Jarosław Bryk acquired the data.

### Conflict of interest statement

The Guest Associate Editor Frederic J. J. Chain declares that, despite being affiliated to the same institution as authors Jaroslaw Bryk and Diethard Tautz, the review process was handled objectively and no conflict of interest exists. The authors declare that the research was conducted in the absence of any commercial or financial relationships that could be construed as a potential conflict of interest.

## Supplementary material

Table S1 **List of all genes on the custom CGH microarray**.

Table S2 **List of all CNVs found. (A)** contains CNVs ordered by sample and genomic position and (B) contains CNVs ordered by Ensembl gene ID affected by CNVs.

Table S3 **List of overlaps of identified CNVs with known Ensembl exons and introns**.

Table S4 **Results of gene ontology analysis**. All significant categories (*p* < 0.05 after FDR correction) are reported.

Table S5 **Overview of CNVs differentiating the two populations**.

Table S6 **Primers used for microsatellite amplification**.

Table S7 **lnRH statistics for microsatellite length heterozygosity**.
